# Bromidotetra­kis­(1*H*-2-ethyl-5-methyl­imidazole-κ*N* ^3^)copper(II) bromide

**DOI:** 10.1107/S1600536811051117

**Published:** 2011-11-30

**Authors:** Sylwia Godlewska, Katarzyna Baranowska, Joanna Socha, Anna Dołęga

**Affiliations:** a Department of Inorganic Chemistry, Faculty of Chemistry, Gdańsk University of Technology, 11/12 G. Narutowicz St., 80233 PL Gdańsk, Poland

## Abstract

The Cu^II^ ion in the title compound, [CuBr(C_6_H_10_N_2_)_4_]Br, is coordinated in a square-based-pyramidal geometry by the N atoms of four imidazole ligands and a bromide anion in the apical site. Both the Cu^II^ and Br^−^ atoms lie on a crystallographic fourfold axis. In the crystal, the [CuBr(C_6_H_10_N_2_)_4_]^+^ complex cations are linked to the uncoordinated Br^−^ anions (site symmetry 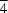) by N—H⋯Br hydrogen bonds, generating a three-dimensional network. The ethyl group of the imidazole ligand was modelled as disordered over two orientations with occupancies of 0.620 (8) and 0.380 (8).

## Related literature

For more copper(II) complexes with bromido and imidazole ligands, see: Godlewska *et al.* (2011 ▶); Hossaini Sadr *et al.* (2004 ▶); Li *et al.* (2007 ▶); Liu *et al.* (2007 ▶); Näther *et al.* (2002**a* ▶,b* ▶). For the alignment of dipoles in crystalline materials, see: Anthony & Radhakrishnan (2001 ▶).
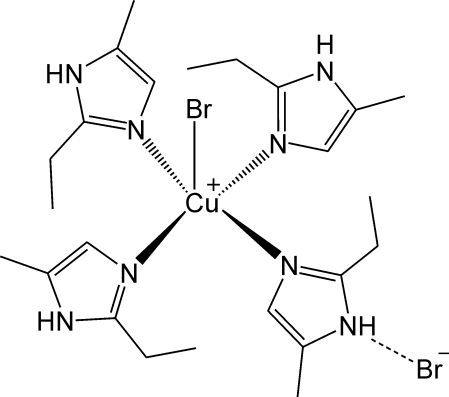


## Experimental

### 

#### Crystal data

[CuBr(C_6_H_10_N_2_)_4_]Br
*M* *_r_* = 664
Tetragonal, 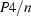

*a* = 14.0961 (4) Å
*c* = 7.5236 (4) Å
*V* = 1494.94 (10) Å^3^
*Z* = 2
Mo *K*α radiation
μ = 3.43 mm^−1^
*T* = 294 K
0.54 × 0.45 × 0.33 mm

#### Data collection

Oxford Diffraction Xcalibur Sapphire2 diffractometer
Absorption correction: analytical [*CrysAlis PRO* (Oxford Diffraction, 2006 ▶), based on expressions derived by Clark & Reid (1995 ▶)] *T*_min_ = 0.248, *T*_max_ = 0.44
5099 measured reflections
1395 independent reflections
898 reflections with *I* > 2s(*I*)
*R*_int_ = 0.025

#### Refinement

*R*[*F* ^2^ > 2σ(*F* ^2^)] = 0.027
*wR*(*F* ^2^) = 0.076
*S* = 0.95
1395 reflections
103 parameters
H-atom parameters constrained
Δρ_max_ = 0.63 e Å^−3^
Δρ_min_ = −0.39 e Å^−3^

### 

Data collection: *CrysAlis PRO* (Oxford Diffraction, 2006 ▶); cell refinement: *CrysAlis PRO*; data reduction: *CrysAlis RED* (Oxford Diffraction, 2006 ▶); program(s) used to solve structure: *SHELXS97* (Sheldrick, 2008 ▶); program(s) used to refine structure: *SHELXL97* (Sheldrick, 2008 ▶); molecular graphics: *ORTEP-3* (Farrugia, 1997 ▶); software used to prepare material for publication: *WinGX* (Farrugia, 1999 ▶).

## Supplementary Material

Crystal structure: contains datablock(s) I, global. DOI: 10.1107/S1600536811051117/hb6533sup1.cif

Structure factors: contains datablock(s) I. DOI: 10.1107/S1600536811051117/hb6533Isup2.hkl

Additional supplementary materials:  crystallographic information; 3D view; checkCIF report

## Figures and Tables

**Table:** 

Cu1—Br1	2.7330 (8)
Cu1—N1^i^	2.029 (2)

**Table:** 

N1^i^—Cu1—N1	89.708 (9)
N1—Cu1—N1^ii^	171.81 (12)

Symmetry codes: (i) 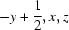; (ii) 
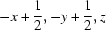
.

**Table 2:** Hydrogen-bond geometry (Å, °)

*D*—H⋯*A*	*D*—H	H⋯*A*	*D*⋯*A*	*D*—H⋯*A*
N2—H2⋯Br2^iii^	0.86	2.63	3.488 (2)	178

Symmetry code: (iii) 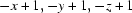.

**Fig. 1.**
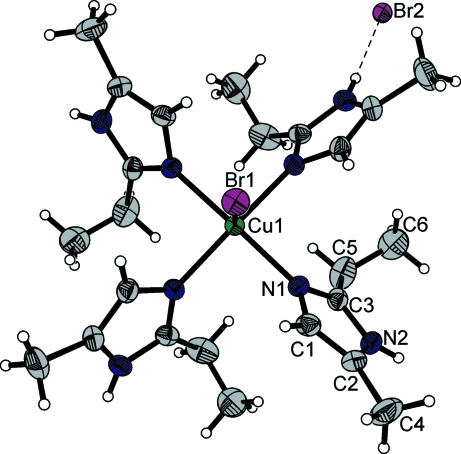
A view of (I), showing displacement ellipsoids drawn at the 30% probability level. Labels are given only for the independent part.

**Fig. 2.**
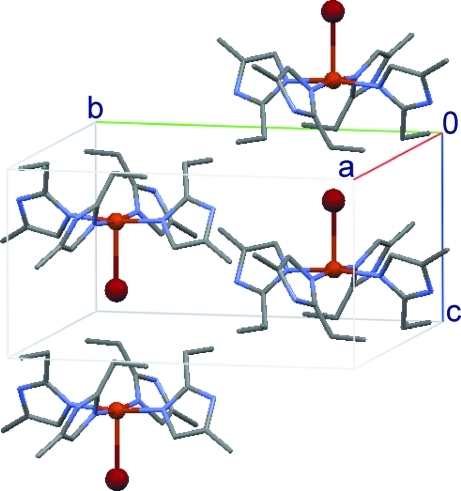
The packing of dipoles in crystals of (I).

## supplementary crystallographic information

### Comment

The composition of (I) is very similar to the bromidotetrakis(1*H*2-isopropylimidazole-κ*N*^3^)copper(II) bromide described previously (Godlewska *et al.*, 2011). However these compounds display substantially different crystal packing. Four NH···Br hydrogen bonds surrounding Br2 in bromidotetrakis(1*H*2-isopropylimidazole-κ*N*^3^)copper(II) bromide are almost planar whereas the corresponding hydrogen bonds in (I) form distorted tetrahedron around Br2 and therefore build a network extending in all three directions in crystal. Complex cations [Cu(C_6_H_10_N_2_)_4_Br]^+^ are dipoles aligned perfectly parallel to *c* axis in a head-to-tail manner (see Fig. 2). The disorder of alkyl substituents is typical of room temperature determinations (*e.g.* Näther *et al.* (2002*a*), *Acta Cryst. E*58, *m*63-*m*64).

The structure of (I) is shown in Fig. 1 and packing diagram of complex dipoles is presented in Fig.2.

### Experimental

The title compound was prepared by adding the solution of 0.223 g (1 mmol) copper(II) bromide in 4 ml of methanol to the solution of 0.496 g (4.5 mmol) 2-ethyl-4(5)-methylimidazole in 2 ml of methanol. After a few days blue prisms were obtained by slow evaporation of solvent from the reaction mixture.

### Refinement

All C–H hydrogen atoms were refined as riding on carbon atoms with methyl C–H = 0.98 Å, methine C–H = 1 Å, aromatic C–H = 0.95 Å and *U*_iso_(H)=1.2 *U*_eq_(C)for aromatic and methine CH and 1.5*U*_eq_(C) for methyl groups. Ethyl group of imidazole was refined as disordered between two positions with occupancies 0.620 (8)/0.380 (8).

### Crystal data

**Table:**

[CuBr(C_6_H_10_N_2_)_4_]Br	*D*_x_ = 1.475 Mg m^−^^3^
*M**_r_* = 664	Mo *K*α radiation, λ = 0.71073 Å
Tetragonal, *P*4/*n*	Cell parameters from 2155 reflections
Hall symbol: -P 4a	θ = 2.7–28.8°
*a* = 14.0961 (4) Å	µ = 3.43 mm^−^^1^
*c* = 7.5236 (4) Å	*T* = 294 K
*V* = 1494.94 (10) Å^3^	Prism, blue
*Z* = 2	0.54 × 0.45 × 0.33 mm
*F*(000) = 678	

### Data collection

**Table:**

Oxford Diffraction Xcalibur Sapphire2 diffractometer	1395 independent reflections
graphite	898 reflections with *I* > 2s(*I*)
Detector resolution: 8.1883 pixels mm^-1^	*R*_int_ = 0.025
ω scans	θ_max_ = 25.5°, θ_min_ = 2.7°
Absorption correction: analytical [*CrysAlis PRO* (Oxford Diffraction, 2006), based on expressions derived by Clark & Reid (1995)]	*h* = −17→10
*T*_min_ = 0.248, *T*_max_ = 0.44	*k* = −17→15
5099 measured reflections	*l* = −9→7

### Refinement

**Table:**

Refinement on *F*^2^	Primary atom site location: structure-invariant direct methods
Least-squares matrix: full	Secondary atom site location: difference Fourier map
*R*[*F*^2^ > 2σ(*F*^2^)] = 0.027	Hydrogen site location: inferred from neighbouring sites
*wR*(*F*^2^) = 0.076	H-atom parameters constrained
*S* = 0.95	*w* = 1/[σ^2^(*F*_o_^2^) + (0.0447*P*)^2^] where *P* = (*F*_o_^2^ + 2*F*_c_^2^)/3
1395 reflections	(Δ/σ)_max_ = 0.004
103 parameters	Δρ_max_ = 0.63 e Å^−^^3^
0 restraints	Δρ_min_ = −0.39 e Å^−^^3^

### Special details

**Table:**

Experimental. CrysAlisPro (Oxford Diffraction Ltd., 2006) Analytical numeric absorption correction using a multifaceted crystal model based on expressions derived by R.C. Clark & J.S. (Clark, R. C. & Reid, J. S. (1995). Acta Cryst. A51, 887-897)
Geometry. All e.s.d.'s (except the e.s.d. in the dihedral angle between two l.s. planes) are estimated using the full covariance matrix. The cell e.s.d.'s are taken into account individually in the estimation of e.s.d.'s in distances, angles and torsion angles; correlations between e.s.d.'s in cell parameters are only used when they are defined by crystal symmetry. An approximate (isotropic) treatment of cell e.s.d.'s is used for estimating e.s.d.'s involving l.s. planes.
Refinement. Refinement of *F*^2^ against ALL reflections. The weighted *R*-factor *wR* and goodness of fit *S* are based on *F*^2^, conventional *R*-factors *R* are based on *F*, with *F* set to zero for negative *F*^2^. The threshold expression of *F*^2^ > σ(*F*^2^) is used only for calculating *R*-factors(gt) *etc*. and is not relevant to the choice of reflections for refinement. *R*-factors based on *F*^2^ are statistically about twice as large as those based on *F*, and *R*- factors based on ALL data will be even larger.

### Fractional atomic coordinates and isotropic or equivalent isotropic displacement parameters (Å^2^)

**Table:**

	*x*	*y*	*z*	*U*_iso_*/*U*_eq_	Occ. (<1)
Br1	0.25	0.25	0.31140 (7)	0.0495 (2)	
Br2	0.75	0.25	0	0.05286 (19)	
Cu1	0.25	0.25	0.67466 (8)	0.0366 (2)	
N1	0.22250 (14)	0.39093 (14)	0.6939 (3)	0.0397 (5)	
N2	0.21196 (15)	0.54003 (15)	0.7653 (3)	0.0486 (6)	
H2	0.2195	0.5922	0.823	0.058*	
C1	0.16846 (19)	0.4385 (2)	0.5685 (4)	0.0481 (7)	
H1	0.141	0.4104	0.4693	0.058*	
C2	0.16140 (19)	0.5299 (2)	0.6100 (4)	0.0520 (8)	
C3	0.24764 (19)	0.4552 (2)	0.8122 (4)	0.0446 (7)	
C4	0.1150 (3)	0.6113 (2)	0.5178 (5)	0.0900 (14)	
H4A	0.0826	0.5888	0.4138	0.135*	
H4B	0.1623	0.6568	0.4836	0.135*	
H4C	0.0702	0.6408	0.5966	0.135*	
C5	0.2915 (9)	0.4385 (17)	0.980 (3)	0.059 (3)	0.620 (8)
H5A	0.2972	0.3709	1.001	0.071*	0.620 (8)
H5B	0.2533	0.4658	1.0744	0.071*	0.620 (8)
C6	0.3952 (5)	0.4870 (5)	0.9776 (9)	0.087 (3)	0.620 (8)
H6A	0.3886	0.5546	0.9672	0.131*	0.620 (8)
H6B	0.4307	0.4632	0.8783	0.131*	0.620 (8)
H6C	0.4279	0.4721	1.0859	0.131*	0.620 (8)
C5A	0.3233 (13)	0.438 (2)	0.966 (4)	0.043 (5)	0.380 (8)
H5A1	0.3232	0.3711	0.9977	0.052*	0.380 (8)
H5A2	0.386	0.4535	0.9218	0.052*	0.380 (8)
C6A	0.3036 (11)	0.4935 (7)	1.1209 (14)	0.123 (6)	0.380 (8)
H6A1	0.3115	0.5595	1.0934	0.184*	0.380 (8)
H6A2	0.3466	0.476	1.2143	0.184*	0.380 (8)
H6A3	0.2395	0.4822	1.1588	0.184*	0.380 (8)

### Atomic displacement parameters (Å^2^)

**Table:**

	*U*^11^	*U*^22^	*U*^33^	*U*^12^	*U*^13^	*U*^23^
Br1	0.0587 (3)	0.0587 (3)	0.0311 (3)	0	0	0
Br2	0.0394 (2)	0.0394 (2)	0.0798 (5)	0	0	0
Cu1	0.0350 (2)	0.0350 (2)	0.0397 (4)	0	0	0
N1	0.0396 (13)	0.0367 (12)	0.0428 (14)	0.0018 (10)	−0.0011 (10)	−0.0029 (10)
N2	0.0519 (15)	0.0364 (13)	0.0576 (17)	−0.0021 (11)	0.0071 (13)	−0.0080 (12)
C1	0.0411 (17)	0.0519 (19)	0.0514 (18)	0.0037 (14)	−0.0084 (14)	−0.0031 (15)
C2	0.0455 (18)	0.0466 (19)	0.064 (2)	0.0092 (14)	−0.0011 (16)	0.0027 (16)
C3	0.0485 (18)	0.0436 (17)	0.0418 (17)	−0.0049 (13)	0.0058 (14)	−0.0022 (14)
C4	0.086 (3)	0.058 (2)	0.127 (4)	0.0231 (19)	−0.030 (2)	0.007 (2)
C5	0.060 (9)	0.070 (5)	0.048 (6)	−0.003 (8)	−0.003 (7)	−0.007 (4)
C6	0.104 (6)	0.079 (4)	0.079 (6)	0.010 (4)	−0.047 (4)	−0.021 (3)
C5A	0.038 (11)	0.060 (8)	0.033 (8)	−0.024 (10)	−0.007 (8)	0.007 (6)
C6A	0.252 (18)	0.065 (7)	0.051 (7)	0.045 (8)	−0.051 (9)	−0.023 (6)

### Geometric parameters (Å, °)

**Table:**

Cu1—Br1	2.7330 (8)	C4—H4A	0.96
Cu1—N1^i^	2.029 (2)	C4—H4B	0.96
Cu1—N1	2.029 (2)	C4—H4C	0.96
Cu1—N1^ii^	2.029 (2)	C5—C6	1.613 (14)
Cu1—N1^iii^	2.029 (2)	C5—H5A	0.97
N1—C3	1.319 (3)	C5—H5B	0.97
N1—C1	1.385 (3)	C6—H6A	0.96
N2—C3	1.344 (3)	C6—H6B	0.96
N2—C2	1.376 (4)	C6—H6C	0.96
N2—H2	0.86	C5A—C6A	1.43 (3)
C1—C2	1.330 (4)	C5A—H5A1	0.97
C1—H1	0.93	C5A—H5A2	0.97
C2—C4	1.492 (4)	C6A—H6A1	0.96
C3—C5	1.42 (2)	C6A—H6A2	0.96
C3—C5A	1.59 (3)	C6A—H6A3	0.96
			
N1^i^—Cu1—N1	89.708 (9)	N2—C3—C5A	125.4 (12)
N1^i^—Cu1—N1^ii^	89.707 (9)	C2—C4—H4A	109.5
N1—Cu1—N1^ii^	171.81 (12)	C2—C4—H4B	109.5
N1^i^—Cu1—N1^iii^	171.81 (12)	H4A—C4—H4B	109.5
N1—Cu1—N1^iii^	89.707 (9)	C2—C4—H4C	109.5
N1^ii^—Cu1—N1^iii^	89.708 (9)	H4A—C4—H4C	109.5
N1^i^—Cu1—Br1	94.10 (6)	H4B—C4—H4C	109.5
N1—Cu1—Br1	94.10 (6)	C3—C5—C6	108.3 (12)
N1^ii^—Cu1—Br1	94.10 (6)	C3—C5—H5A	110
N1^iii^—Cu1—Br1	94.10 (6)	C6—C5—H5A	110
C3—N1—C1	106.0 (2)	C3—C5—H5B	110
C3—N1—Cu1	132.03 (19)	C6—C5—H5B	110
C1—N1—Cu1	122.00 (18)	H5A—C5—H5B	108.4
C3—N2—C2	108.9 (2)	C6A—C5A—C3	112.1 (18)
C3—N2—H2	125.5	C6A—C5A—H5A1	109.2
C2—N2—H2	125.5	C3—C5A—H5A1	109.2
C2—C1—N1	110.5 (3)	C6A—C5A—H5A2	109.2
C2—C1—H1	124.7	C3—C5A—H5A2	109.2
N1—C1—H1	124.7	H5A1—C5A—H5A2	107.9
C1—C2—N2	105.1 (2)	C5A—C6A—H6A1	109.5
C1—C2—C4	132.0 (3)	C5A—C6A—H6A2	109.5
N2—C2—C4	122.8 (3)	H6A1—C6A—H6A2	109.5
N1—C3—N2	109.5 (2)	C5A—C6A—H6A3	109.5
N1—C3—C5	127.0 (10)	H6A1—C6A—H6A3	109.5
N2—C3—C5	122.8 (10)	H6A2—C6A—H6A3	109.5
N1—C3—C5A	124.3 (12)		

Symmetry codes: (i) −*y*+1/2, *x*, *z*; (ii) −*x*+1/2, −*y*+1/2, *z*; (iii) *y*, −*x*+1/2, *z*.

### Hydrogen-bond geometry (Å, °)

**Table:**

*D*—H···*A*	*D*—H	H···*A*	*D*···*A*	*D*—H···*A*
N2—H2···Br2^iv^	0.86	2.63	3.488 (2)	178

Symmetry codes: (iv) −*x*+1, −*y*+1, −*z*+1.
